# *Streptococcus pneumoniae* carriage and antibiotic use in children in southwestern Uganda

**DOI:** 10.4102/jphia.v16i1.880

**Published:** 2025-03-14

**Authors:** Birungi Mutahunga, Nahabwe Haven, Orikushaba I. Magezi, James Mubangizi, Yusufu Kuule, Peter R. Scull, Frank M. Frey

**Affiliations:** 1 Department of Health, Kanungu District Local Government, Kanungu, Uganda; 2 Department of Infectious Diseases, University of California Davis, Davis, United States of America; 3 Community Health and Batwa, Bwindi Community Hospital, Kanungu, Uganda; 4 Clinical Laboratory, Bwindi Community Hospital, Kanungu, Uganda; 5 Department of Geography, Colgate University, Hamilton, United States of America; 6 Department of Biology, Colgate University, Hamilton, United States of America

**Keywords:** antimicrobial, caretaker, household survey, prevalence, *Streptococcus pneumonia*

## Abstract

**Background:**

Acute respiratory infection is a significant health threat in children under the age of 5 years in Uganda and can be caused by *Streptococcus pneumoniae*.

**Aim:**

This study documents caretaker behaviour in seeking treatment for suspected acute respiratory infection in children and estimates the prevalence of *S. pneumoniae* in healthy and sick children.

**Setting:**

The study was carried out in the catchment region of Bwindi Community Hospital, encompassing the sub-counties of Kanyantorogo, Kayonza and Mpungu in rural southwestern Uganda.

**Methods:**

This cross-sectional study was conducted from January 2023 to August 2023. Caretakers answered questions about the child’s illness, symptoms, sources of treatment and medicines administered. Nasopharyngeal swabs were collected from children and cultured to identify *S. pneumoniae* using standard microbiological methods. Analyses were conducted using SPSS and ArcPro GIS.

**Results:**

Roughly half of the 422 families sampled reported that the child was ill within the past 2 weeks, the vast majority with symptoms consistent with a possible acute respiratory infection. Most (80%) sought treatment outside the home at a private or public health facility or drug shop. Regardless of treatment source, antibiotics (primarily amoxicillin) were administered 56% of the time. The prevalence of *S. pneumoniae* was 34% and positively associated with household density, household size and the number of children in the household.

**Conclusion:**

This study documents a high carriage rate of *S. pneumoniae* in the region and documents a high rate of antibiotic use in the region.

**Contribution:**

This study provides evidence to support a wider assessment of *S. pneumoniae* carriage and the potential for antibiotic resistance.

## Introduction

*Streptococcus pneumoniae* (*S. pneumoniae*) readily colonises the nasopharynx of children under 5 years, and adults, and while usually asymptomatic it can sometimes spread to surrounding tissue or invade the bloodstream, causing pneumococcal diseases ranging from a mild upper respiratory infection to acute otitis media, severe pneumonia or bacterial meningitis.^1^ In 2015, there were over nine million estimated cases of *S. pneumoniae* infection in children under 5 years globally.^2^ However, the estimated number of deaths in children under 5 years because of *S. pneumoniae* infection declined from 600 000 in 2000 to 294 000 in 2015, owing to the reintroduction of the pneumococcal conjugate vaccine (PCV) in 129 countries at the time.^2^

A 10-valent PCV with a three-dose schedule (PCV10) was incorporated into the Uganda National Immunisation Programme in 2014.^3^ Following a slow start because of supply chain logistics,^3^ Uganda achieved an estimated 90% coverage nationwide,^4,5^ with 96% coverage through three doses in the Kigezi region of Uganda^6^ where this study took place. However, high vaccination coverage has not necessarily corresponded with a decrease in the prevalence of *S. pneumoniae* or the pneumococcal disease burden. Using local surveillance data from the Kigezi region in southwestern Uganda, Busoga region in eastern Uganda and Tooro region in mid-western Uganda, the pneumococcal disease burden in 2020 was slightly lower (40%) compared to what it was pre-vaccination (60%), and highest in the Kigezi region of Uganda (52%).^7^ The pre-vaccination prevalence of *S. pneumoniae* in children under 5 years ranged between 59% and 75% in southwestern Uganda,^8^ eastern Uganda^9,10^ and Kampala.^11^ In an urban study done in Kampala where 98% of children under 5 years were vaccinated with PCV10, the nasopharyngeal carriage rate of *S. pneumoniae* in 2019 was 46%,^12^ and in a rural study conducted from 2019 to 2021 in western Uganda neighbouring Kibale National Park, the carriage rate was 78%.^13^

Acute respiratory infection is currently a leading disease condition among children under 5 years outpatient attendances in Uganda, second only to malaria, and is a leading cause of children under 5 years admission and children under 5 years mortality along with malaria and neonatal conditions.^14^ The 2022 Demographic and Health Survey showed that 8% of children under 5 years in Uganda had a suspected acute respiratory infection, with 84% of those with a suspected acute respiratory infection seeking treatment outside the home.^6^

The Kigezi region of southwestern Uganda encompasses the districts of Kabale, Kanungu, Kisoro and Rukungiri; and the Kanungu district is divided into 2 counties and 18 sub-counties with a total of 102 parishes and 527 villages. Our study was focussed on the Kanungu district within the catchment area of Bwindi Community Hospital (BCH), which encompasses 101 villages organised into 13 parishes in the sub-counties of Kanyantorogo, Kayonza and Mpungu. In this article, we report on the behaviours of caretakers as it relates to seeking treatment for children under 5 years acute respiratory infections and accessing medicines, and also on factors associated with the prevalence of *S. pneumoniae* in children under 5 years.

## Research methods and design

### Study setting and sample size

**FIGURE 1:**
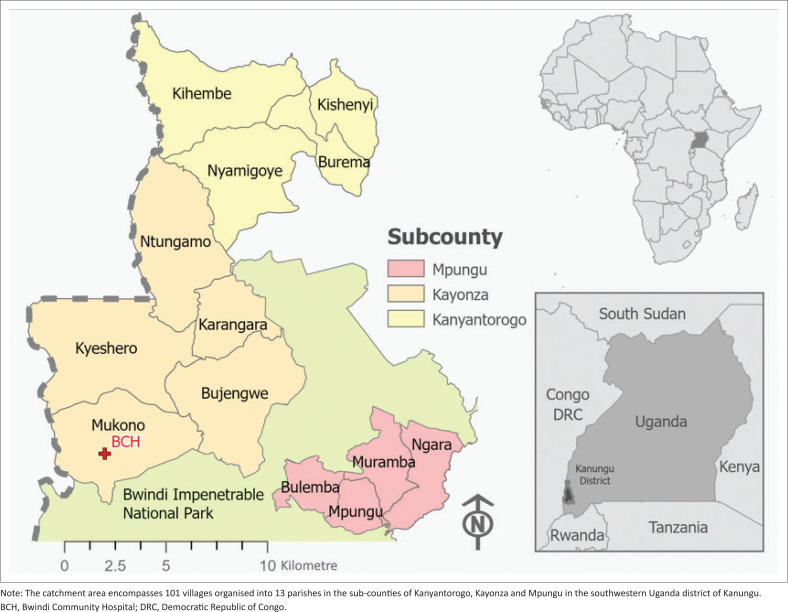
Study area showing the catchment region of Bwindi Community Hospital.

This study was conducted between January 2023 and August 2023 in the catchment region of BCH, which encompasses 101 villages organised into 13 parishes in the sub-counties of Kanyantorogo, Kayonza and Mpungu in the southwestern Uganda district of Kanungu (Figure 1). Bwindi Community Hospital is a private, not-for-profit facility that was established under the Diocese of Kinkiizi, Church of Uganda in 2003, and has since grown to a 155-bed hospital offering surgical, obstetric, orthopaedic, and paediatric sub-specialties with an associated training wing at Uganda Nursing School – Bwindi and Uganda College of Health Sciences. Bwindi Community Hospital has a staff of approximately 200 including doctors, clinical officers, nurses, midwives, other health workers and support staff, and cares for more than 120 000 people living in their catchment area and beyond. The hospital has been consistently ranked as one of the top-performing hospitals in all of Uganda by the Uganda Protestant Medical Bureau and USAID Uganda’s STAR Southwest programme. The BCH Diagnostics Laboratory, established in 2013, performs over 500 tests per day in the areas of biochemistry, haematology, serology and microscopy.

We used two approaches to calculate the sample size. Firstly, we used the standard formula for prevalence studies (see Equation 1):

$$n=\left[Z^{2}P(1-P)\right]/d^{2}$$ [Eqn 1]

where *Z* is the *Z* statistic for a level of confidence, *P* is the expected prevalence and *d* is the precision.^15,16^ The prevalence of *S. pneumoniae* in Ugandan children has been previously estimated as 77%,^8^ 56%,^9^ 59%^10^ and 62%.^11^ Using a rounded average for prevalence at 60%, a 95% confidence level and 5% precision yielded a sample size of 369. Secondly, we used Epitools^17^ to determine the sample size needed to estimate a true prevalence with an imperfect test. We also used an estimated prevalence of 60%, a 95% confidence level and a 5% precision value. Sensitivity and specificity values for detecting *S. pneumoniae* using optochin disc sensitivity assays have both been estimated as 100%^18^ and 99% and 98%, respectively.^19^ We used a 99% value for both sensitivity and specificity of the optochin disc assay to yield a sample size of 385. We decided to use the larger of the two sample size calculations and added a 10% buffer to set our final target sample size at 424 participants.

### Study and survey design

At the time of the study, BCH had records in their database of approximately 10 000 children under 5 years distributed across the 13 parishes as follows: Bujengwe (9%), Burema (5%), Buremba (5%), Karangara (6%), Kihembe (9%), Kishenyi (5%), Kyeshero (13%), Mpungu (6%), Mukono (12%), Muramba (5%), Ngaara (5%), Ntungamo (14%) and Nyamigoye (7%). To identify potential participants for inclusion in the study, we randomly selected households from the database in accordance with this distribution for a proportionate-based stratified sampling design so that our sample would be representative of the distribution of children under 5 years in the catchment region. Community health nurses visit every village at least once every 2 months to document births and deaths, perform immunisations, check on maternal and child health, administer treatments and coordinate patient transport to BCH. We prepared a list of households organised by the village for possible inclusion in the study with the head of the household and children under 5 years listed along with a household global positioning system (GPS) coordinate when possible. On the day they were scheduled to visit a particular village, community health nurses would pick up this prepared list along with surveys and swabs (more later in the article); and then as their workday permitted, they would seek to locate the households identified for possible inclusion in the study, obtain informed consent, administer the survey and collect a nasopharyngeal swab. We kept track of the sampling progress weekly, and when the threshold for each parish was reached we stopped sampling villages within that parish. In the end, the distribution of our realised sample was representative of the distribution of children under 5 years in the catchment region: Bujengwe (11%), Burema (6%), Buremba (8%), Karangara (11%), Kihembe (6%), Kishenyi (8%), Kyeshero (10%), Mpungu (7%), Mukono (9%), Muramba (5%), Ngaara (4%), Ntungamo (11%) and Nyamigoye (5%).

The World Health Organization (WHO) has published guidance on assessing access to medicines at the household^20^ level, and like other studies on household medicinal use in Uganda (e.g., ^10,21,22,23,24^), we drew specific questions from this guidance and also consulted an Uganda Ministry of Health Survey related to access and use of medicines.^25^ Our survey instrument first collected household demographic information (age and sex of caretaker, age and sex of children under 5 years participant, household size) and then asked: if the child had been sick within the past 2 weeks^20^ (WHO Q3), if the child was still sick, the type of health problems or symptoms^20^ (WHO Q7), if any home remedies (traditional medicines) were used, if any medicines present in the home were used, if treatment was sought outside the home^20^ (WHO Q9), what the source of that treatment was^20^ (WHO Q10), if treatment was recommended^20^ (WHO Q11), what medicines were recommended^20^ (WHO 12), how much the medicines cost^20^ (WHO Q13), whether the child took the medicine as directed^20^ (WHO Q15) and whether any medicine was left over. The survey was written in English and translated into Rukiga (the local dialect of Luganda) and piloted and revised twice.

### Study procedures

Community health nurses were trained in best practices for administering the caretaker survey^20^ and the proper collection of nasopharyngeal specimens in accordance with WHO guidelines.^26^ Upon reaching a pre-identified household for potential inclusion in the study, the community health nurse would read the informed consent document to the caretaker and obtain their signature (or thumb-print if they could not read or write) if they affirmed consent, and then proceed to administer the survey, recording data on a handwritten form. The caretaker was defined as the adult who identified as the primary caregiver to the child. The household ID number and GPS coordinates from the BCH database, where available, were pre-filled on the survey form to be used for each pre-identified household, and the community health nurse checked these GPS coordinates using a Garmin e-trex 10 (Garmin, United States [US]) noting any discrepancies. Finally, a nasopharyngeal sample was collected from the under 5 year old child with a single sterile flexible mini-tip nylon flocked swab (Copan, US) and immediately inserted into a sterile tube containing a 5 mL column of Aimes agar gel transport media with charcoal (Oxoid, United Kingdom [UK]). Tubes were labelled with the household ID number, date and name of the community health nurse, and returned to the BCH Diagnostics Laboratory as soon as possible (usually within 8 h of sample collection). Upon return to BCH, community health nurses entered the data from each survey into a secure Google Form that we created for this study. Photographs of the informed consent form and survey form were taken and uploaded into a secure Google Form, while hard copies of each were organised into binders and stored in separate binders in separate locations. Only B.M., F.M.F. and P.R.S. had access to the data files.

### Laboratory procedures

Sample swabs in their labelled tubes were delivered to the BCH Diagnostics Laboratory where they were recorded on an intake sheet. Swabs were streaked on separate 5% blood agar plates with an optochin disc (Oxoid, UK) placed in the first streak area and then incubated at 37 °C under 5% CO_2_ for 24 h. A reference strain of *S. pneumoniae* (ATCC 49619) was used as quality control. *S. pneumoniae* was identified according to WHO guidelines,^26^ including an inhibition zone greater than 14 mm around the optochin disc, the presence of small, grey, alpha-haemolytic colonies with central depression at maturity and gram-positive diplococci that were catalase negative. Replicate liquid cultures of colonies were preserved at −80 °C for future serotyping and antibiotic resistance analyses.

### Spatial analysis

Community health nurses recorded geo-location data from each consenting household using a Garmin e-trex 10 GPS (Garmin, US). In addition, and in order to characterise the relative proximity of each household to different healthcare facilities, we visited and recorded the location of the four health centre III (three public, one private) and eight health centre II (two public, six private) facilities in the BCH catchment region. In Uganda, health centre III facilities are led by a senior clinical officer with an outpatient clinic, maternity ward and basic laboratory, and health centres II facilities are led by an enrolled nurse with an outpatient clinic and a focus on treating common diseases and antenatal care. We also visited and recorded the location of 45 drug shops in the BCH catchment region, less than half of which were registered with the Uganda National Drug Authority (*n* = 18/45, 40%).

ArcPro GIS (ESRI, US) was used to determine the elevation of each household. We also measured the Euclidian distance between each household and each of the different healthcare facilities, as well as the closest drug shop. Using all georeferenced households, we used the kernel density tool in ArcPro to calculate the household density (average number of households per square kilometre) across the region.

### Data management and statistical analysis

The survey data entered by the community health nurses was independently verified using photographs of the forms (F.M.F and P.R.S.). These data were downloaded to an Microsoft Excel spreadsheet and coded independently (F.M.F and P.R.S.). Laboratory results were downloaded to an Microsoft Excel spreadsheet. These data were merged into one file for analysis in Statistical Package for the Social Sciences (SPSS). Descriptive statistics of frequencies were conducted and compared using Chi-squared analysis. Bivariate analysis of individual characteristics in relation to *S. pneumoniae* carriage was analysed using Chi-squared analysis for child age, child sex and sub-county, and one-way analysis of variance (ANOVA) for household size and the number of children under the age of 18 years in the household. We used logistic regression to evaluate risk factors for pneumococcal carriage. Factors with a *p* < 0.10 in the bivariate analysis were used in the multivariable model and odds ratios (ORs), 95% confidence intervals (CIs) and *p*-values were calculated. We used a threshold value of 0.05 as our Type I error rate to evaluate *p*-values.

### Ethical considerations

Study procedures were approved by the Colgate University Institutional Review Board (FR-F19-01), the Mbarara University of Science and Technology (MUST) Research Ethics Board (01/01-20) and the Uganda National Council for Science and Technology (HS2625ES). Verbal consent to allow data collection from community members was obtained from village leaders before data collection took place, and the informed consent form was made available and read aloud in Rukiga to caretakers. Data collection only started after the adult caretaker had given signed consent or, if unable to read or write, a thumb-print consent. Binders containing hard-copy informed consent, survey and laboratory results forms were housed separately from each other. The identities of the participants were anonymised during data analysis. Community health nurses and laboratory personnel were only able to upload the information they collected to the secure Google folders, and only the principal investigators on the grant (B.M., F.M.F. and P.R.S.) had access to the full data set.

## Results

### Characteristics of the study population

We were able to collect survey and sample data from 422 households. Caretakers responding were mostly male with an average age of 35 years (standard deviation [s.d.] = 11, median = 34, range = 18–80; Table 1). Female and male children were evenly represented with an average age of 33 months (s.d. = 15, median = 36, range = 6–60; Table 1). Households contained 5.4 individuals on average (s.d. = 2.1, median = 5, range = 2–12; Table 1), with an average of 2.7 individuals under the age of 18 years (s.d. = 1.8, median = 2, range = 1–9; Table 1). Caretaker age did not vary across sub-counties, but caretaker sex did with Mpungu having a more balanced distribution (*χ*^2^ = 16.05, degree of freedom [*df*] = 2, *p* < 0.001; Table 2). Child age did not vary across sub-counties, but child sex did with Mpungu having more of a male bias than the other two sub-counties (*χ*^2^ = 8.67, *df* = 2, *p* = 0.013; Table 2).

**TABLE 1:** Demographics of the study population.

Variable	Frequency	
	*n*	%
**Caretaker age (*n* = 406)**		
30 years and under	120	30
31–59 years	269	66
60 years and over	14	4
**Caretaker sex (*n* = 416)**		
Female	136	33
Male	280	67
**Child age (*n* = 422)**		
12 months and under	34	8
13–36 months	174	41
36–60 months	214	51
**Child sex (*n* = 422)**		
Female	200	47
Male	222	53
**Household size (*n* = 422)**		
2	27	6
3	55	13
4	75	18
5	75	18
6	76	18
7	51	12
8	29	7
9	14	3
10	9	2
11	5	1
12	6	1
**Number of children < 18 years (*n* = 422)**		
1	151	36
2	65	15
3	74	18
4	61	14
5	39	9
6	21	5
7	4	1
8	6	1
9	1	0

**TABLE 2:** Demographics of study population across sub-counties.

Category	Kanyantorogo		Kayonza		Mpungu	
	*n*	%	*n*	%	*n*	%
Children under-5 years in database	-	26	-	53	-	21
Children under-5 years in sample	100	24	221	52	101	24
**Caretaker age (*n* = 406)**						
30 years and under	29	30	60	29	31	31
31–59 years	65	68	140	67	64	63
60 years and over	2	2	9	4	6	6
**Caretaker sex (*n* = 416)**						
Female	24	24	63	29	49	49
Male	76	76	152	71	52	51
**Child age (*n* = 422)**						
12 months and under	7	5	19	9	8	8
13–36 months	40	40	93	42	41	41
36–60 months	53	53	109	49	52	51
**(*n* = 422)**						
Female	52	52	113	51	35	35
Male	48	48	108	49	66	65

Note: Caretaker age: *χ*^2^ = 2.05, *df* = 4, *p* = 0.727. Caretaker sex: *χ*^2^ = 16.05, *df* = 2, *p* < 0.001. Child age: *χ*^2^ = 0.51, *df* = 4, *p* = 0.973. Child sex: *χ*^2^ = 8.67, *df* = 2, *p* = 0.013.

The average number of individuals in the household varied among sub-counties (Kanyantorogo = 4.97 ± 2.28 (s.d.), Kayonza = 5.38 ± 2.03 (s.d.), Mpungu = 5.91 ± 2.15 (s.d.); *F* [2,419] = 4.00, *p* = 0.007), as did the average number of children under 18 years of age in the household (Kanyantorogo = 2.53 ± 1.64 (s.d.), Kayonza = 2.48 ± 1.68 (s.d.), Mpungu = 3.49 ± 1.83 (s.d.); *F* [2, 419] = 12.96, *p* < 0.001).

### Survey analysis

Caretakers reported that 216 out of 422 (51%) of the children had been sick in the 2 weeks prior to sample collection, that 135 out of 216 (32%) were still sick at the time of sample collection, and that 190 out of 216 (88%) had symptoms consistent with a possible acute respiratory infection (e.g., runny nose, cough and difficulty breathing). The frequency of children being sick within the past 2 weeks did not differ among sub-counties (Kanyantorogo = 44%, Kayonza = 52%, Mpungu = 57%; 𝜒^2^ = 3.25, *df* = 2, *p* = 0.197), but Kayonza had a higher proportion of children sick at the time of sample collection compared to the other sub-counties (Kanyantorogo = 57%, Kayonza = 71%, Mpungu = 49%; 𝜒^2^ = 8.76, *df* = 2, *p* = 0.013). The frequency of children with symptoms consistent with a possible acute respiratory infection did not vary among sub-counties (Kanyantorogo = 84%, Kayonza = 92%, Mpungu = 83%; 𝜒^2^ = 4.18, *df* = 2, *p* = 0.124).

Caretakers reported that 30 out of 216 (14%) of the children who had been sick within the past 2 weeks did not receive treatment at home or outside of the home. Caretakers reported that 39 out of 216 (18%) of the children who had been sick in the 2 weeks prior to sample collection received some form of treatment at home. The frequency of home treatment did not vary across sub-county (Kanyantorogo = 11%, Kayonza = 20%, Mpungu = 19%; 𝜒^2^ = 1.69, *df* = 2, *p* = 0.431). The most common home treatment for these 39 children was receiving medicinal herbs (*n* = 32) or paracetamol (*n* = 20), and very few children received leftover antibiotics in the household (*n* = 8, mostly amoxicillin).

Caretakers reported that 174 out of 216 (81%) of the children who had been sick in the 2 weeks prior to sample collection received treatment outside of the home. Of these 174 children, 27 had initially received some form of treatment at home before seeking treatment outside of the home and 147 had only received treatment outside of the home. Children in Kayonza were most likely to receive treatment outside of the home (88%), followed by children in Mpungu (75%) and Kanyantorogo (68%; 𝜒^2^ = 9.14, *df* = 2, *p* = 0.010). Overall, it was most common to visit private healthcare facilities (45%), followed by drug shops (41%) and public healthcare facilities (29%), with very few caretakers seeking treatment from neighbours (1%) or friends (1%). Most caretakers sought treatment outside the home from one source only (88%), and of those seeking treatment from multiple sources (*n* = 21), the most common combination was visiting a drug shop and a private healthcare facility (67%).

We looked at how the source of treatment outside the home varied among sub-counties in two ways. Firstly, we looked at how the total number of visits (*n* = 195) per sub-county was distributed among public, drug shop and private facilities; this analysis included individuals who had sought more than one source of treatment (*n* = 21). Secondly, we looked at how the total number of visits for caretakers seeking only one source of treatment (*n* = 153) per sub-county was distributed among public, drug shops and private facilities. In both cases, the same picture emerged with caretakers in Kanyantorogo primarily visiting private facilities, caretakers in Kayonza primarily visiting drug shops and caretakers in Mpungu primarily visiting public facilities (Table 3).

**TABLE 3:** Treatment outside the home across sub-counties.

Category	Kanyantorogo		Kayonza		Mpungu	
	*n*	%	*n*	%	*n*	%
**Individuals seeking one or more sources of treatment (*n* = 195; 174 individuals)**					
Public	8	23	22	19	20	43
Drug shop	6	18	49	43	12	25
Private	20	59	43	38	15	32
*n*	34	-	114	-	47	-
**Individuals seeking only one source of treatment (*n* = 153)**					
Public	8	31	19	22	16	41
Drug shop	2	8	37	42	9	23
Private	16	61	32	36	14	36
*n*	26	-	88	-	39	-

Note: Individuals seeking one or more sources of treatment: *χ*^2^ = 17.32, *df* = 4, *p* = 0.002. Individuals seeking only one source of treatment: *χ*^2^ = 15.83, *df* = 4, *p* = 0.003.

When seeking treatment outside the home, some form of medicinal treatment was usually recommended by the provider (99%) and that treatment was usually taken (98%). Most commonly, some form of antibiotic was given as treatment (59%); and amoxicillin was the most frequently administered antibiotic (77%) compared to trimethoprim/sulfamethoxazole (14%), metronidazole (6%), cefotaxime (4%), ampicillin (3%), erythromycin (2%) and gentamicin (2%). More than one antibiotic was administered in < 1% of cases. Antibiotics were administered similarly to those presenting with symptoms of a possible acute respiratory infection (60%) and those presenting with other symptoms (50%) (𝜒^2^ = 0.53, *df* = 1, *p* = 0.466).

There were 149 individuals who sought treatment outside the home from a single source and took the recommended medicinal treatment and the majority (56%) were given an antibiotic. Antibiotics were recommended at similar rates among public health facilities (59%), drug shops (56%) and private health facilities (53%) (𝜒^2^ = 0.33, *df* = 2, *p* = 0.848). In addition to antibiotics, paracetamol was a commonly recommended medicinal treatment (59%), and less frequent were antimalarials (18%), antihistamines (17%), piritex syrup (12%) and anthelmintics (4%).

The amount of money in Ugandan currency (UGX) that caretakers paid when seeking treatment outside the home varied widely (mean = 6700 UGX ± 800 UGX [s.e. {standard error}], range: 0–64 000 UGX). The amount of money caretakers paid was similar in Kanyantorogo (mean = 6700 UGX ± 800 UGX [s.e.]), Kayonza (mean = 6600 UGX ± 1900 UGX [s.e.]) and Mpungu (mean = 7100 UGX ± 1100 UGX [s.e.]; *F* [2,163] = 0.285, *p* = 0.752). Caretakers paid much less at public facilities (mean = 2500 UGX ± 1400 UGX [s.e.]) compared to drug shops (mean = 8100 UGX ± 1100 UGX [s.e.]) and private facilities (mean = 8400 UGX ± 1600 UGX [s.e.]; *F* [2,142] = 4.55, *p* = 0.012).

### Prevalence of nasopharyngeal *S. pneumoniae*

Overall, 145 out of 422 (34%) of the samples were positive for *S. pneumoniae* and the prevalence did not vary across child age groups (12 months and under = 35%, 13–35 months = 40%, 36–60 months = 30%; 𝜒^2^ = 4.06, *df* = 2, *p* = 0.131) or child sex (female = 35%, male = 34%; 𝜒^2^ = 0.07, *df* = 1, *p* = 0.793).

Prevalence varied among sub-counties with Kanyantorogo (27%) and Kayonza (25%) having a much lower prevalence than Mpungu (61%; 𝜒^2^ = 43.08, *df* = 2, *p* < 0.001). Within sub-county, the prevalence was lower in male children than female children in Kanyantorogo (female = 37%, male = 17%, 𝜒^2^ = 5.00, *df* = 1, *p* = 0.025) and similar in Kayonza (female = 27%, male = 24%, 𝜒^2^ = 0.18, *df* = 1, *p* = 0.672) and Mpungu (female = 60%, male = 62%; 𝜒^2^ = 0.04, *df* = 1, *p* = 0.835). We excluded children 12 months or under because of sample size limitations and found that in Kanyantorogo, the prevalence was higher in the 13–35 month age group compared to the 36–60 month age group (13–35 months = 45%, 36–60 months = 13%; 𝜒^2^ = 11.72, *df* = 1, *p* < 0.001), and the prevalence between these age groups was more similar in Kayonza (13–35 months = 26%, 36–60 months = 25%; 𝜒^2^ = 0.03, *df* = 1, *p* = 0.866) and Mpungu (13–35 months = 66%, 36–60 months = 58%; 𝜒^2^ = 0.64, *df* = 1, *p* = 0.422).

Children positive for *S. pneumoniae* were from larger households compared to children negative for *S. pneumoniae* (negative = 5.25 ± 0.13 [s.e.], positive = 5.71 ± 0.18 [s.e.], *F* [1, 420] = 4.45, *p* = 0.036) and also were from households with a greater number of children under the age of 18 years (negative = 2.55 ± 0.10 [s.e.], positive = 3.08 ± 0.16 [s.e.]; *F* [1, 420] = 8.64, *p* = 0.003).

The prevalence of *S. pneumoniae* was slightly higher in children who had been sick in the 2 weeks prior to sample collection (*n* = 84/216, 39%) compared to healthy children (*n* = 61/206, 30%; 𝜒^2^ = 4.02, *df* = 1, *p* = 0.045). The prevalence of *S. pneumoniae* was higher in children who were still sick at the time of sample collection (*n* = 60/135, 44%) compared to those who had been sick in the 2 weeks prior to sample collection but were not currently sick (*n* = 24/81, 30%; 𝜒^2^ = 4.68, *df* = 1, *p* = 0.031). When only children with symptoms of a possible acute respiratory infection were included (*n* = 190/216, 88%), the prevalence of *S. pneumoniae* was higher in children that were still sick at the time of sample collection (*n* = 58/129, 45%) compared to those that had been sick in the 2 weeks prior to sample collection but were not currently sick (*n* = 16/61, 26%; 𝜒^2^ = 6.11, *df* = 1, *p* = 0.013).

The prevalence of *S. pneumoniae* was not different between children who received home treatment and those who did not (𝜒^2^ = 0.01, *df* = 1, *p* = 0.952), between those who sought treatment outside the home and those who did not (𝜒^2^ = 0.06, *df* = 1, *p* = 0.814), among those that sought treatment at public, drug shop or private facilities (𝜒^2^ = 1.16, *df* = 2, *p* = 0.559), and among those that received antibiotic treatment compared to those that did not (𝜒^2^ = 1.30, *df* = 1, *p* = 0.255).

### Spatial analysis

**FIGURE 2:**
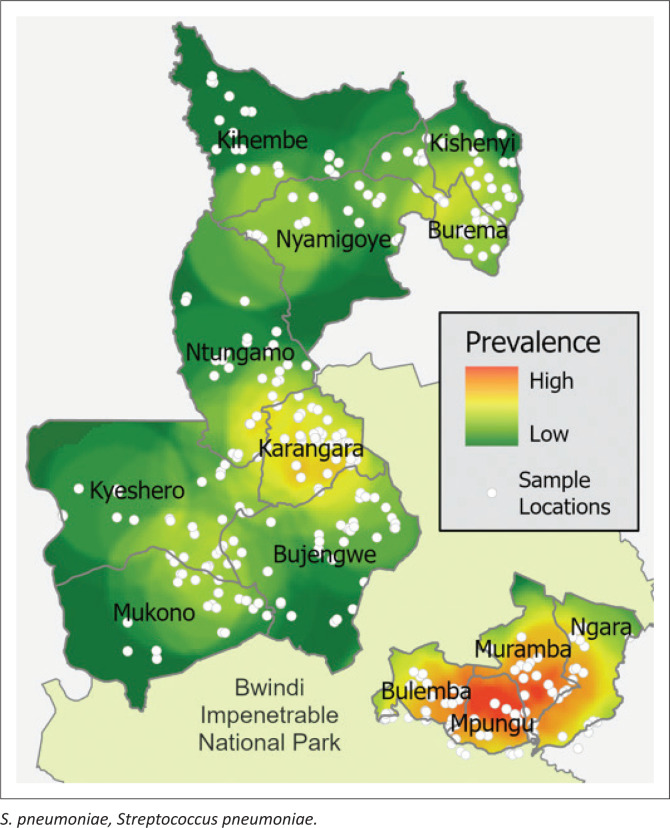
Study area showing the distribution of samples (that we have location data for) with an overlaid heat map of the prevalence of *S. pneumoniae* ranging from low (0%) to high (100%).

We were able to collect location data from 356 out of 422 (84%) households and these data were evenly spread out among sub-counties (Kanyantorogo *n* = 92/100 [92%]; Kayonza *n* = 172/221 [78%]; Mpungu *n* = 92/101 [92%]). The prevalence of *S. pneumoniae* among households that we have location data for (*n* = 130/356, 37%; Figure 2) was similar to the overall prevalence of *S. pneumoniae* in the entire data set (*n* = 145/422, 34%). Samples positive for *S. pneumoniae* were from households that were at higher elevations (negative = 1393 metre [m] ± 13 [s.e.], positive = 1513 m ± 23 [s.e.]; *F* [1, 354] = 22.84, *p* < 0.001) and closer to health centre II (negative = 3114 m ± 120 [s.e.], positive = 2745 m ± 132 [s.e.]; *F* [1, 354] = 3.91, *p* = 0.049) and health centre III facilities (negative = 3341 m ± 134 [s.e.], positive = 2889 m ± 138 [s.e.]; *F* [1, 354] = 4.88, *p* = 0.028). There was no difference in proximity to drug shops (negative = 1159 m ± 42 [s.e.], positive = 1065 m ± 57 [s.e.]; *F* [1, 354] = 1.83, *p* = 0.177). Samples positive for *S. pneumoniae* were from households at higher density (negative = 43 households/km^2^ ± 2 [s.e.], positive = 52 households/km^2^ ± 2 [s.e.]; *F* [1, 354] = 9.18, *p* = 0.003).

There were 153 caretakers that sought treatment outside the home from a single source and we had location data for 130 of these households. We calculated the distance from each of these households to the nearest public, drug shop and private facility and then asked what percentage of the time they sought treatment at the facility closest to their home. In 54 out of 130 (42%) cases, caretakers sought treatment at the facility that was closest to their home (Kanyantorogo = 5 out of 26 [19%], Kayonza = 35 out of 68 [51%], Mpungu = 14 out of 36 [39%]) and in 76 out of 130 (58%) cases caretakers sought treatment at a facility that was not closest to their home.

### Risk factors for carriage of *S. pneumoniae*

To assess risk factors for the carriage of *S. pneumoniae* bivariate and multivariable logistic regression was performed (Table 4). In the bivariate analysis, we found that the following factors were positively associated with the odds of pneumococcal carriage: household size (*p* = 0.037), the number of children under the age of 18 years in the household (*p* = 0.004), being sick within the past 2 weeks (*p* = 0.045) and at the time of sample collection (*p* = 0.032), distance from health centre II (*p* = 0.050) and health centre III facilities (*p* = 0.029), and household density (*p* = 0.004). We did not use household size or being sick at the time of sample collection in the multivariable analysis because of their obvious correlations with the number of children under the age of 18 years in the household and being sick within the past 2 weeks, respectively. In the multivariate analysis, we found that the strongest predictor of pneumococcal carriage was household density (*p* = 0.005) followed by the number of children under the age of 18 years in the household (*p* = 0.034) and being sick within the past 2 weeks (*p* = 0.041). There was little multicollinearity in this analysis (number of children and sick within the past 2 weeks = −0.03; number of children and household density = −0.02; household density and sick within the past 2 weeks = 0.02).

**TABLE 4:** Factors associated with *S. pneumoniae* carriage.

Variable	Category†	Unadjusted‡			Adjusted		
		OR	95% CI	*p*	OR	95% CI	*p*
Sex	Female	Ref.§	-	-	-	-	-
	Male	0.95	0.63–1.42	0.793	-	-	-
Age	< 12 months	Ref.	-	-	-	-	-
	13–35 months	1.21	0.56–2.60	0.634	-	-	-
	36–60 months	0.79	0.37–2.68	0.527	-	-	-
Household size	Continuous	1.11	1.01–1.21	0.037	Excluded	-	-
Number < 18	Continuous	**1.18**	**1.06–1.33**	**0.004**	**1.14**	**1.01–1.29**	**0.034**
Sick past 2 weeks	No	Ref.	-	-	-	-	-
	Yes	**1.51**	**1.01–2.27**	**0.045**	**1.59**	**1.02–2.49**	**0.041**
Sick now	No	Ref.	-	-	Excluded	-	-
	Yes	1.90	1.06–3.41	0.032	-	-	-
Sub-county	Kanyantorogo	Ref.	-	-	-	-	-
	Kayonza	0.92	0.54–1.57	0.753	-	-	-
	Mpungu	4.30	2.37–7.80	< 0.001	-	-	-
Elevation	Continuous	1.01	1.00–1.01	< 0.001	-	-	-
Dist. Drug Shop	Continuous	1.00	0.99–1.01	0.178	-	-	-
Dist. HC II	Continuous	1.00	1.00–1.00	0.050	-	-	-
Dist. HC III	Continuous	1.00	1.00–1.00	0.029	-	-	-
Household density	Continuous	**1.01**	**1.00–1.02**	**0.003**	**1.01**	**1.00–1.02**	**0.005**

Note: Bold values in the table highlight the predictors with *p* < 0.05 from the multivariable regression.

Dist. distribution; HC, health centre.

† , Bivariate logistic regression analyses with continuously distributed variables are indicated as ‘Continuous’, otherwise the values of the categories are given;

‡ , Odds ratios (ORs), 95% confidence intervals (95% CIs), and *p*-values (*p*) are given for each bivariate logistic regression analysis (unadjusted) and the multivariable logistic regression analysis (adjusted);

§ , We have indicated the reference category (Ref.) for each categorical bivariate logistic regression analysis.

## Discussion

In our study, about 50% of the children under 5 years sampled had been sick within the past 2 weeks and the vast majority had symptoms consistent with a possible acute respiratory infection. About 20% of caretakers used some form of treatment at home, most often medicinal herbs and paracetamol and very infrequently antibiotics left over in the household. About 80% of caretakers sought treatment outside the home and about 60% of the time they received an antibiotic for their child, regardless of whether they visited a public or private health facility or a drug shop, and most often this antibiotic was amoxicillin. This high rate of antibiotic distribution to children under 5 years is consistent with studies in northern Uganda,^21,23,24^ eastern Uganda,^10,27^ and the capital city of Kampala.^22,27^ However, the actual antibiotic administered varies widely in the published literature from Uganda. Three studies showed a high rate of amoxicillin distribution in northern^21,24^ and eastern^27^ Uganda. In contrast, trimethoprim/sulfamethoxazole was commonly administered in a study from eastern Uganda^10^ and metronidazole was most commonly administered in a study from Kampala.^27^ In two studies,^22,23^ amoxicillin and trimethoprim/sulfamethoxazole were administered at the same high rate.

In Uganda, drug shops are authorised to sell over-the-counter medications (class-C) drugs but not antibiotics.^28^ In our study, drug shops administered antibiotics more than 50% of the time (and mostly amoxicillin), which is perhaps not surprising given the lack of regulation enforcement in Uganda; for example, a study from central Uganda that found over 90% of drug shops were selling antibiotics.^29^ In addition, our study found that caretakers were provided with antibiotics at the same rate at which they received paracetamol across public and private facilities and drug shops. This is troublesome because, as others have noted,^21,22,23,24,27,29^ the majority of suspected acute respiratory infections in children under 5 years are viral and the mis-administration of antibiotics promotes the evolution of resistance, resulting in possible poor clinical outcomes and wastes resources.

Our study also showed that caretakers do not generally visit the provider that is closest to their household and that there were geographic differences in where caretakers sought treatment. In Kanyantorogo, caretakers primarily visited private health centre II facilities over public health centre III facilities or drug shops. In Mpungu, caretakers primarily visited the public health centre III facility over the private health centre II facility or drug shops. In Kayonza, caretakers primarily visited drug shops for treatment over the public and private health centre II and health centre III facilities. These patterns are not driven by proximity and could be related to socioeconomic factors (caretakers paid more than 3 times as much at private facilities and drug shops compared to public facilities), perceived wait times, prior experience or some other cultural or social factor.^23,24^

The overall prevalence of *S. pneumoniae* in this study (34%) was lower than studies conducted in Uganda before the widespread implementation of PCV10^8,9,10,11^ and lower than studies conducted after the introduction of this vaccine.^12,13^ However, the prevalence of *S. pneumoniae* in Mpungu was relatively unchanged (61%) from pre-vaccination estimates in Uganda despite the reported high coverage (96%) through three doses of PCV10 in the region.^6^ Future work will compare the serotype distribution among these regions and to those included in PCV10 to shed light on why the prevalence of *S. pneumoniae* in Kanyantorogo and Kayonza was much lower than in Mpungu and to gauge the effectiveness of PCV10. Predictably, while the carriage of *S. pneumoniae* was slightly higher in children under 5 years who had been sick compared to healthy children and there was also a relatively high carriage rate among healthy children (30%), a result that is consistent with other recent work in Uganda.^12,13^ Commensal carriage of *S. pneumoniae* in children under 5 years is thought to be a reservoir of transmission to the vulnerable age groups of infants and the elderly; for example, one recent study of agricultural workers in the United States has suggested that exposure to children under 5 years and household overcrowding results in a 1.5 times greater chance of contracting *S. pneumoniae* in adults.^30^ Consistent with that finding, our study shows that children under 5 years positive for *S. pneumoniae* were from larger households, from households with a greater number of children under the age of 18 years and from areas of greater household density compared to children negative for *S. pneumoniae*.

### Limitations

As a cross-sectional study, the work presented here is limited by measuring caretaker behaviour and *S. pneumoniae* prevalence at one point in time and an inability to infer causal relationships. Caretaker responses in the presence of a BCH nurse could have been biased and caretaker responses could have suffered from imperfect memories. Although we took great care to generate a spatially representative sample of the BCH catchment region and observed participation rates similar to expected participation rates, it is possible that our sample is not representative of the population. We used standard microbiological techniques to identify *S. pneumoniae*, rather than molecular methods such as real-time polymerase chain reaction polymerase chain reaction (PCR) assays targeting *lytA* and multi-locus sequence typing (MLST), which may have greater reliability in making positive identifications and differentiating from various viridian species.

## Conclusion

This study shows that, in the sub-counties of Kanyantorogo, Kayonza and Mpungu in rural southwestern Uganda, caretakers of children under 5 years with symptoms of acute respiratory infection commonly seek treatment outside the home and commonly receive antibiotics from public and private health facilities and drug shops in the region. Caretakers did not primarily visit the closest source of treatment to their household and there were geographic differences in where treatment was sought, perhaps related to social or cultural factors and prior experience. The prevalence of *S. pneumoniae* was higher than expected given the widespread coverage of PCV10. Household density, household size and the number of children in the household were all identified as risk factors for pneumococcal carriage.
